# Inflammatory cytokines dysfunction in autism spectrum disorder

**DOI:** 10.1192/j.eurpsy.2021.571

**Published:** 2021-08-13

**Authors:** A. Ben Othman, H. Slama, E. Cherif, M. Azaiez, H. Gharsallah

**Affiliations:** 1 Child And Adolescent Psychiatry, Military Hospital of Instruction of Tunis, Tunis, Tunisia; 2 Anesthesiology And Intensive Therapy, Military Hospital of Instruction of Tunis, Tunis, Tunisia

**Keywords:** autism spectrum disorder, neurodevelopment, neuroinflammation, inflammatory cytokines

## Abstract

**Introduction:**

Autism spectrum disorder (ASD) is a common neurodevelopmental disorder. Its underlying causes and pathophysiologies remain unclear. Recent data support the potential involvement of neuroinflammation in the onset of this disorder.

**Objectives:**

The aim of our study was to investigate the potential link between ASD and inflammatory mediators.

**Methods:**

This descriptive study was conducted among ASD outpatients followed-up at the child and adolescent psychiatry department in the Military Hospital of Instruction of Tunis. Blood samples were collected for inflammatory cytokines dosage, notably the interleukin 1β (IL-1β), interleukin 6 (IL-6) and the Tumor Necrosis Factor α (TNF-α) immunodosage.

**Results:**

Twenty-four patients were included in this study, aged between four and ten years old (mean age= 6,55 years; minimum=4; maximum=10 years). Our sample was mainly represented by male patients (95,6%). TNF-α plasmatic levels were high (>5pg/mL) among all of our sample with a mean of 11,6 pg/mL (minimum= 6,87; maximum=17,7 pg/mL; standard deviation= 3,52 pg/mL), suggesting abnormal peripheral blood mononuclear cells response. However, IL-1β and IL-6 plasmatic levels were relatively normal.

**Conclusions:**

An immune response dysregulation was detected in our sample. Multiple clinical and experimental studies investigated the implication of inflammatory cytokines in neurodevelopmental disruption. Their results, however, remain controversial and limited by small samples. Further studies need to be done in order to investigate the neuroimmunological factors linked with ASD.

